# Hazardous Waste Disposal in Stromatolitic-Limestone Terrain and Hexavalent Chromium Contamination in Chhattisgarh State, India

**DOI:** 10.5696/2156-9614-10.27.200907

**Published:** 2020-08-25

**Authors:** Alka Banchhor, Madhurima Pandey, Meena Chakraborty, Piyush Kant Pandey

**Affiliations:** 1 Department of Applied Chemistry, Bhilai Institute of Technology, Durg, Chhattisgarh, India; 2 Department of Chemistry, Government Naveen College Bori, Durg, Chhattisgarh, India; 3 Institute of Health Management Research, IIHMR University, Jaipur, Rajasthan, India

**Keywords:** hazardous waste, landfill, hexavalent chromium, solid waste, leachate, water pollution

## Abstract

**Background.:**

Hexavalent chromium-containing waste from chromite ore processing is a major environmental health hazard due to its high toxicity. There have been instances of improper and unsafe disposal of this waste, leading to environmental health hazards.

**Objectives.:**

The objective of the present study was to identify the cause of yellow colored water discharge and reported health issues in nearby residents and cattle. In addition, it investigated the improper disposal of chromite ore processing residue (COPR), a hazardous waste, in an abandoned quarry in stromatolitic-limestone terrain in central-east India.

**Methods.:**

Standard methods of analysis of water and wastewater were used for the analyses of variables, including hexavalent chromium (Cr(VI)), pH, sulfate (SO_4_^2−^), chlorine (Cl^−^), total hardness, calcium (Ca(II)), magnesium (Mg(II)), alkalinity and sodium (Na(I)) with proper sampling, quality assurance, and quality control protocols. Onsite Cr(VI) was analyzed using a chromium testing kit, and in the laboratory by atomic absorption spectrophotometry.

**Results.:**

Large-scale contamination of surface and groundwater was noted due to the migration of hexavalent chromium-contaminated yellow colored leachate. High levels of hexavalent chromium were noted in the samples. The maximum Cr(VI) concentration observed was 1050 mg/L in leachate, 22 mg/L in surface water and 0.26 mg/L in the groundwater sample. Acute health effects were noted in cattle and by residents who consumed the highly contaminated water.

**Conclusions.:**

A large volume of discharge of hexavalent chromium contamination from the COPR landfill was found, indicating the absence of containment features in the design (double high-density polyethylene liners, clay, leachate collection). Disposal of COPR in an abandoned limestone mine is inadvisable. The highly fractured stromatolitic-limestone environment at the study site was found to offer almost no resistance to the mobilization of Cr(VI) due to the absence of organic or eukaryotic deposition in the stromatolitic environment. It was also noted that the drainage pattern of the area facilitates a possible translocation of contaminated discharge to the nearby river system. Nearby residents were unaware of the adverse impacts of the contaminated leachates and were using the contaminated water for bathing, washing, etc. Applicable Indian governmental regulations regarding the construction of hazardous waste landfills were found to be insufficient with respect to the use of inactive limestone mines as landfill sites.

**Competing Interests.:**

The authors declare no competing financial interests.

## Introduction

Chromium (Cr) is an active oxidation-reduction element with two commonly encountered valence states, Cr(III) and Cr(VI), which are the most stable forms found in the environment.^1^ However, these two oxidation states exhibit markedly different physical and chemical behavior and toxicology, which is manifested in significantly different environmental mobility and health concerns.

Chromium compounds play an essential role in any industrial economy and are widely used in metallurgy, the chemical industry, military, textiles, and machinery. Among the chromium salts, consumption of basic chromium sulfate and chromium oxide accounts for more than half of total consumption. Other widely used compounds include chromium chloride and the chromium salts of organic acids. Significant uses include chrome plating, manufacturing of dyes and pigments, leather tanning and wood preservation. These compounds are also used for drilling mud, as rust and corrosion inhibitors, textiles, and toner for copy machines.

Chromium compounds include chromates, dichromates, and other derivatives like chromic oxides, chromium sulfate, etc. The process of mining chromium ore (mainly chromite) and processing to produce various compounds generate a considerable amount of chromium-rich solid waste, and liquid effluent and atmospheric emissions may contain Cr chiefly as Cr(III) or Cr(VI) species.

Due to the toxicity of Cr(VI)-bearing substances, the pollution problem caused by the residue from metallurgical operations or manufacturing of chromium compounds has become a matter of worldwide concern.^2^ At many worldwide locations, industries have disposed of hazardous waste in ways which are convenient to their self-interests, such as illegal dumpsites, at the expense of the environment and human health. These dump sites are the primary source of Cr contamination and permanent damage to groundwater systems.

After the much publicized controversy of hexavalent chromium contamination of drinking water in the Hinkley community in California due to industrial operations, the issue of chromium contamination and its attendant health effects remain highly controversial.^3^,^4^ The World Health Organization considers Cr(VI) to be a Group 1 carcinogen to humans. The guideline for drinking water sets the maximum permissible concentration limit at 50 μg/L of chromium (total).^5^ The United States Environmental Protection Agency (USEPA) recognizes Cr as a toxic element, but has not set down a rule for Cr(VI). The drinking water standard includes only total chromium (Cr(T)) with a maximum contaminant level of 100 μg/L.^6^

**Abbreviations**
COPR: Chromite ore processing residue
TH: Total hardness
USEPA: United States Environmental Protection Agency

Water can be exposed to waste dumps, either in engineered secure landfills or ordinary municipal landfills, from rainwater percolation or groundwater flow. Depending on the protective strength of the landfill, water can lead to leaching of soluble inorganic and organic fractions of the waste. The liquid effluent generated is called leachate, which can undergo translocation from the landfill site.^7^ Thus, leachates are fluids generated by the release of excess water from solid waste, and by seepage of rainwater through strata of solid waste that is basically in a state of decay.^7^,^8^ Groundwater contamination due to leachate from landfills has been recognized since 1984.^9^–^11^ Previous studies have shown that the scale of this threat depends on the concentration and toxicity of contaminants in leachates, type, and permeability of geological strata, water table depth and the direction of groundwater flow.^12^ Leachate management is a critical factor in landfill management.^13^,^14^ Inadequate securing of landfills containing hazardous substances and their improper operation are recognized as potential health hazards.^15^

There have been reported instances of hexavalent chromium transport due to large-scale groundwater flow in river basins, hence any large scale discharge of Cr(VI) has the potential to reach groundwater as well as rivers draining from the contaminated basin.^16^ Chromium and other heavy metals have reportedly been released at many locations.^17^ High levels of chromium contamination (275 mg/L) of groundwater in the vicinity of tannery waste landfill sites have been reported in Ranipet in the Vellore District of Tamil Nadu, India.^18^,^19^ Dhakate and Singh investigated groundwater contamination at a chromite ore waste dump site in India and found Cr(VI) levels up to 0.45 mg/L.^20^ Municipal landfill leachate monitoring studies from the Czech Republic have reported a high level of chromium and other heavy metals.^21^ Attempts have also been made to conduct tracer studies to detect subsurface chromium contamination and identification of proper waste disposal sites for Cr(VI) wastes.^22^

However, regulatory and public pressure on these industries is compelling them to dispose of wastes in landfills, instead of open dumping, as is prevalent in some areas of the world. The present study addresses Cr(VI) contamination of different environmental matrices including soil, surface water, and groundwater in the vicinity of a chromium waste disposal site in a landfill situated in an abandoned limestone quarry.

## Methods

**Figure 1:**
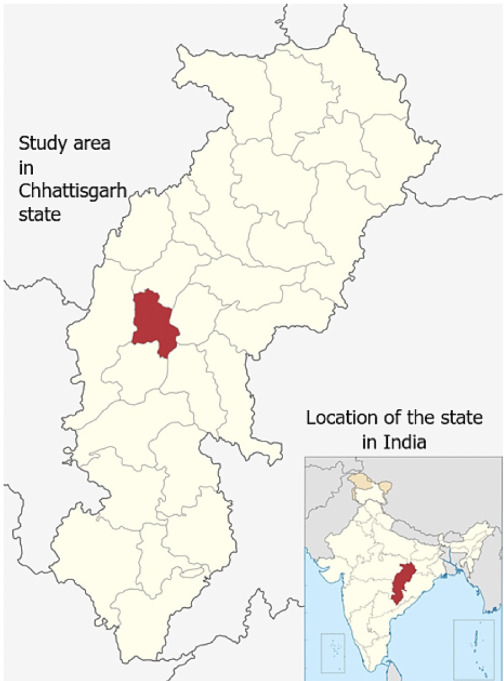
Study area in Chhattisgarh state in India

The study location lies in the vicinity of a hazardous waste landfill constructed in a former limestone quarry in the Nadini-Khundini village of the Durg district in the Chhattisgarh state of India. The Durg district (20°23′-22°02′ N and 80°48′-81°57′ E) lies in the basin-plains of Chhattisgarh. The Shivnath River, a major tributary of the Mahanadi river system, drains from most areas in the district *(Figure 1).* Limestone is the major mineral resource of the district, with an estimated 550 million tons of cement grade to ballast furnace grade limestone reserves in the area. The Bhilai steel plant mines a significant reserve of about 200 million tons of blast furnace grade limestone in the nearby town of Nandini. The area, comprised of villages such as Nadini-Khundini, Meresara, Semaria, Achholi, Ghotwani, Matargota, is being extensively mined for cement grade limestone for use in the cement plant and as paving material.

### Sampling methodology

**Figure 2:**
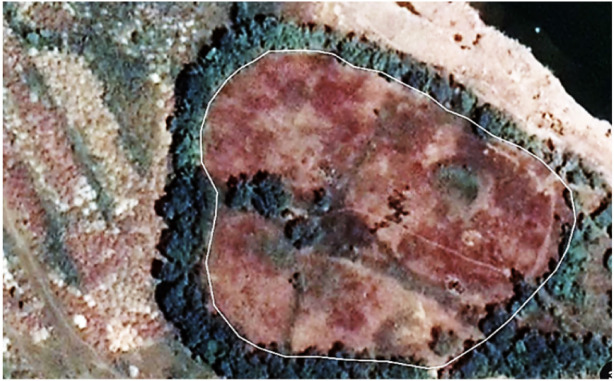
Chromite ore processing residue waste landfill and the sampling area. (Map data: Google, Maxar Technologies)

The sampling locations were distributed around the landfill site *(Figure 2),* and the details of the sampling locations are given in Table 1. The sampling was focused on a landfill located at 21°38′ N- 81°38′ E. This landfill, an abandoned limestone quarry, was used for dumping chromite ore processing residues (COPR).

**Table 1:** Characteristics of Sampling Sites

**Samples No.**	**Sampling area**	**Elevation (m from mean sea level)**	**Distance from the landfill source (m)**	**Nature of sampled source**
1	Sampling area 1	285–280	5–500	Seepage discharge around the landfill site
2	Sampling area 2	279–277	501–1000	Surface flowing water and ponds
3	Sampling area 3	276–250	1001–5000	Pond and hand pumps

The landfill quarry is situated at a height of 280 feet above mean sea level and is the highest location in the sampled region *(Table 1).* Thus, this landfill quarry is a continuous source of leachate from the onset of the monsoon season in the month of May. Seepage from this quarry has been observed throughout most of the year with variation in the magnitude of the discharge, which is highest in the monsoon season and lower in the summer season.

Sampling was carried out as extensively as possible in the first circle (Sampling area 1) around the landfill site. Samples of soil, surface water, and groundwater were collected from the surrounding area in a grab sampling pattern while trying to maintain a sampling grid of appropriate distance. Around 55 samples were collected from sampling area 1, and 46 samples were collected from sampling area 2 and 29 samples were collected from sampling area 3. Figure 3 indicates the sampled locations. The samples included water seeping from hanging walls, surface seepages, water bodies or existing shallow borewells (depth <10 meter).

**Figure 3A:**
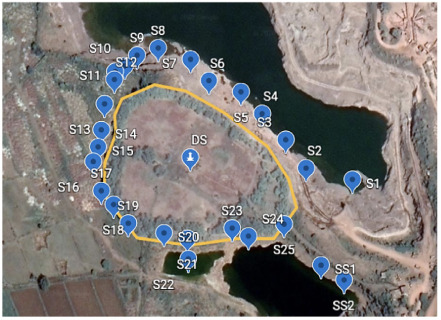
Sampling area 1. Abbreviations: DS, Dumpsite; S, sampling points in sampling area 1. Map data: Google, Maxar Technologies

**Figure 3B:**
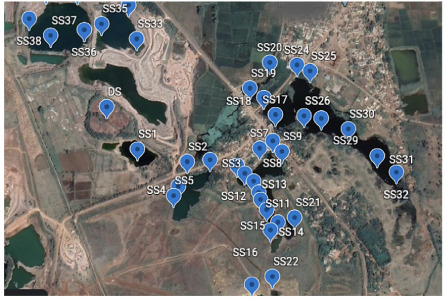
Sampling area 2. Abbreviations: DS, Dumpsite; SS, Sampling points in sampling area 2. Map data: Google, Maxar Technologies

**Figure 3C:**
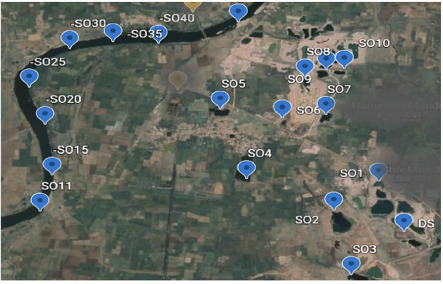
Sampling area 3. Abbreviations: SO, Sampling points in sampling area 3; -SO15, sampling points 11–15; -SO20, sampling points 16–20; -SO25, sampling points 21–25; -SO30, sampling points 26–30; -SO35, sampling points 31–35; -SO40, sampling points 36–40. Map data: Google, Maxar Technologies

Sampling area 1 has an elevation of 285 to 280 m above sea level and is the highest region in the sampled area. The sampling points were mainly natural seepage points originating from the hanging walls of the landfill. Within a distance of about 500 m from the landfill, about 55 samples were collected in the periphery of the landfill. Sampling area 2 was an area approximately 1 km from the landfill with an elevation between 279 to 277 m. This area has many ponds and seepage discharge points, which were sampled to generate 46 samples. Sampling area 3 was an area 1 km to 5 km from the landfill. This area had many regular hand pumps and ponds, situated at an approximate elevation of 276 to 250 m. Around 29 samples were collected from this area.

For quality assurance/quality control, water samples were collected in pre-washed plastic bottles, and soil samples were collected in dry polythene bags. No preservation was done, to maintain the prevailing oxidation state and the pH/redox potential conditions in the collected samples. The samples were refrigerated (<4°C) in the laboratory and analyzed as soon as possible.

### Laboratory analysis

Quality control and quality check protocols consisted of the use of standard samples, analysis of blanks, duplicate analysis of every sample and inter-laboratory calibration checks. We adopted a laboratory independent quality control program by submitting duplicate field blanks and spiked samples.

### Analysis of chromium and hexavalent chromium

Both onsite and off-site analyses were conducted on the collected samples. The Cr(VI) was analyzed both onsite and in the laboratory. Onsite Cr(VI) was analyzed using AQUANAL-plus chromium (Merck, Germany, range 0.005 – 0.1 mg/L). In the laboratory, total chromium was analyzed using an atomic absorption spectrophotometer (Varian, FS-240). The analysis protocol used was as documented in a US Occupational Safety and Health Administration publication titled “Metal and Metalloid Particulates in Workplace Atmospheres (Atomic Absorption)” method number OSHA ID-121.^23^

In the flame atomic absorption spectrophotometer analysis of chromium, a fuel-rich mixture of acetylene and nitrous oxide was used, and the measurement of absorption was carried out at 357.9 nm. Each sample was aspirated in the flame at least two times, and the average value was recorded. The minimum detection limit of the method was 0.04 μg/L. The stock solution of chromium was prepared by using analytical grade chromium trioxide (1.923 g) and was dissolved in water and acidified to pH 2 with pure and concentrated nitric acid. Calibration standards were prepared fresh every day by appropriate dilution of the stock solution. The calibration curve was cross-checked after every ten samples with appropriate use of calibration blanks.

### Analysis of hexavalent chromium colorimetrically

The concentration of Cr(VI) was determined colorimetrically by ultraviolet-visible spectrophotometry following the USEPA 7196A method as previously described.^24^ In this method, the acidic solution of diphenylcarbazide reagent reacts very sensitively with hexavalent chromium to form a red colored stable complex which can be measured quantitatively at 540 nm. In the absence of any interfering radicals such as molybdenum, vanadium, and mercury (as was the case in the present study) the method becomes specific to hexavalent chromium. The minimum detection limit of the method was 50 μg/L.

In the regular analysis method, an aliquot of the sample was taken in a volumetric flask and 2.0 mL diphenylcarbazide was added to it. The pH of the solution was brought to 2±0.5 by adding sulfuric acid (10% vol/vol)_,_ and the volume was made up to the volumetric mark. After about ten minutes, the absorbance was measured at 540 nm with a standard laboratory spectrophotometer following the usual calibration procedure.

For quality control, the certified reference material “Chromium (VI) - Soil certified reference material” (CRM041) from Sigma-Aldrich (CRM041, Supelco, Chromium VI – Soil certified reference material) was used. The reported certified Cr(VI) content of this certified reference material was (86.5 ± 2.91 μg/g).

Standard methods of analysis of water and wastewater by the American Public Health Association were used for the rest of the analyses.^25^ The parameters (pH, oxidation reduction potential, conductivity, total dissolved solids, and salinity) were analyzed onsite and in the lab by using a Benchtop Multiparameter Meter (Thermo-Fisher).

### Geology of the study area

The landfill in the present study is located in a former limestone quarry in the Nadini-Khundini village of Dhamdha block. The landfill was created by a privately-owned chrome chemical company located in the light industrial area of Bhilai in the Durg district of Chhattisgarh state. State authorities permitted the landfill and operations began in an abandoned limestone quarry. The total quantity of disposed waste has not been specified, but it has been calculated that a significantly large quantity of waste generated from COPR has been dumped at the study location.

Topographically, the Nandini-Khundini area is slightly undulating, with ground levels varying from 270 to 290 m above mean sea level. Red soil covers the mineralized limestone area with a clay overburden at a thickness varying from a fraction of meter to about 7 m, with an average thickness of soil of 4 m. The area is sub-arid in climate and receives a large amount of rainfall during the monsoon season (annual rainfall 1288 mm). Due to the topographical features and high monsoonal rainfall, many indistinct streams run through the region following the slopes north and northwest.

Geologically, a typical stromatolitic-limestone terrain topography is predominant. The study area contains large limestone mineralized beds which are horizontal to sub-horizontal (maximum dip ten degrees towards north). In the limestone deposit area of the mines, the bedding plane joints are found predominantly in the second to third bench along with numerous weak joints prevailing all over the area. The area is devoid of major geotectonic disturbances. The presence of columnar branching stromatolites indicates the studied area once had a shallow water marine environment with gentle slopes at the bottom. A protected tidal environment is best suited for such growth. The well-laminated nature of the limestone deposit rules out a deep-sea origin of rocks in the study area. Carbonate sedimentation in the study area can thus be expected to have been formed primarily from the chemical and biochemical process that has occurred in the clear, shallow, warm water marine environment.

## Results

The study location is situated around an underground waste disposal site for COPR. Chromite ore processing residue was found to be produced in a factory situated in the industrial area of Bhilai in Chhattisgarh state where chromite ore is treated with lime (CaO) and soda ash (Na_2_CO_3_) to produce sodium chromate by reaction as in Equation 1 below:

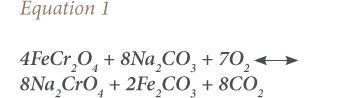



The COPR waste generated was reported to have a varied concentration of products depending on the ore composition and extraction parameters.

In the very first visit to the study location by the research team, yellow colored seepages were found to be coming from various pores and fissures. The chemical analysis of the yellow colored water confirmed a very high level of Cr(VI) contamination in the leachate from the landfill site. Out of the 130 sampled locations, 91 locations showed the presence of substantial amounts of hexavalent chromium *(Table 2).* The high values of Cr(VI) (maximum 22 mg/L) in the seepages were the first indication that the supposedly secure landfill had failed to keep wastes and rainwater separated. It was further noted that the landfill did not have an engineered system for collecting leachate and treating it before discharge.

**Table 2:** Chromium Positive Samples in the Study Area

**Sampling area**	**Total samples collected**	**Chromium positive samples**	**Maximum chromium(VI) mg/L**	**Zone characterization based on the results**
**Sampling area 1**	55	50	22	Primary contamination zone (diagonal distance ~500 m)
**Sampling area 2**	46	35	18.1	Secondary contamination zone (diagonal distance ~1000 m)
**Sampling area 3**	29	6	2.1	Peripheral contamination zone (~5 km)

**Figure 4:**
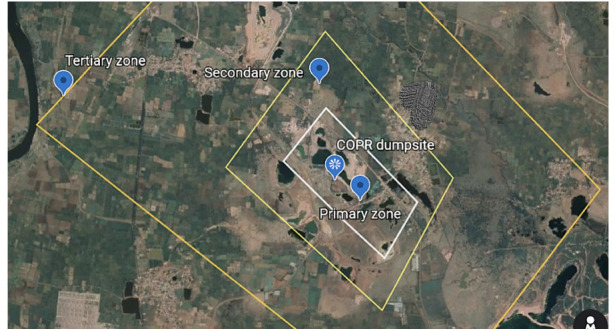
Primary and secondary contamination zone around the waste landfill. (Map data: Google, Maxar Technologies)

The landfill is situated at a high point in the topography *(Figure 2).* This can cause greater percolation of rainwater in the landfill, leading to inflow of water through the columns of waste and thus possible leaching of soluble components from the waste. As expected, the study site was found to be profusely discharging yellow colored liquid from numerous outlets, both from the veins and ore body along the dip and the stromatolite channels in the limestone bedrock in the rainy season. These discharges were found to coincide with the full onset of monsoon rains (July) and continued until the end of the post-monsoon season (December-January). Leachate discharges have led to the creation of a primary contamination zone (diagonal distance ~500 m), secondary contamination zone (diagonal distance ~1000 m) and peripheral zone (extending up to ~ 5 km with a river boundary) as shown in Figure 4. Chemical analysis of the discharge showed that the landfill constructed at this location in the Durg district was generating large amounts of leachate discharge with a high concentration of hexavalent chromium.

### Rainwater-groundwater seepage through limestone quarries

**Figure 5:**
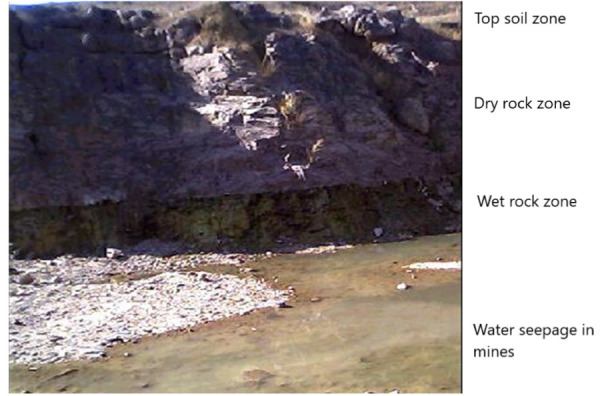
Summer season photograph of a limestone quarry depicting the zonation of dry and water-saturated zones

In the study area, it was further observed that a considerable amount of groundwater seepage took place in limestone mines, even in the dry summer season *(Figure 5).* This is expected, as limestone zones are generally rich in groundwater availability due to percolation of rainwater.

**Figure 6:**
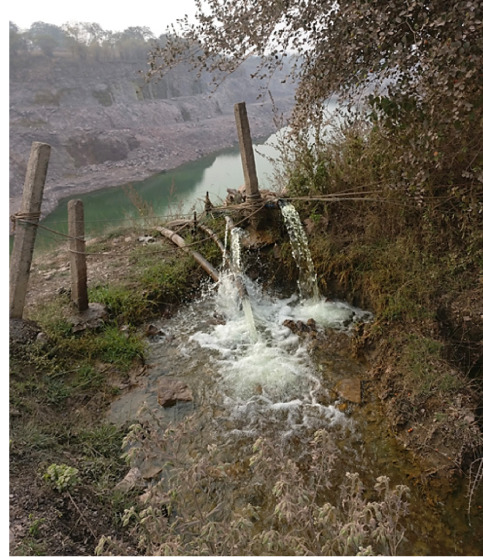
Contaminated water being discharged through heavy duty mine dewatering pumps from an active limestone quarry causing secondary contamination plumes

The studied landfill is situated at the highest elevation in the local topography. High rainfall and elevation have made this landfill an almost perennial source of contaminated seepage through the numerous discharge points in the laminar limestone strata. There are many adjoining limestone mines which are being actively mined. Thus, there are many exposed facies receiving contaminated seepage from the landfill as well as groundwater inflow. Therefore, for continuous mining operation the nearby mines need to pump out the accumulating water *(Figure 6).* This is being done by using mine dewatering pumps to pump out the percolating water. The pumped-out water from nearby mines is discharged onto open fields. Many mine water discharges were found to be contaminated with high levels of hexavalent chromium. Thus, this mine dewatering water was found to be a major source of secondary contamination of nearby surface and groundwater sources.

The concentrated landfill leachate, mine dewatering and contaminated groundwater plume are the principal causes of the groundwater and surface water contamination in the study location. The pond shown in Figure 7 is a result of such contamination where the yellow color landfill discharge accumulation can be seen. The soil and surface water samples around the landfill site have high level of Cr(VI). A visible crystallization of chromium contamination could be seen in the path of flow of the contaminated water *(Figure 8).* The deposited yellow colored precipitate is a mixture of CaCrO_4_, calcium aluminochromate (3CaO•Al_2_O_3_•CrO_4_), tribasic calcium chromate (Ca_3_(CrO_4_)_2_) and sodium chromate (Na_2_CrO_4_).^26^ The industry where this waste originated produced sodium chromate as an intermediary for making other chromium products used for tanning and other uses.

**Figure 7:**
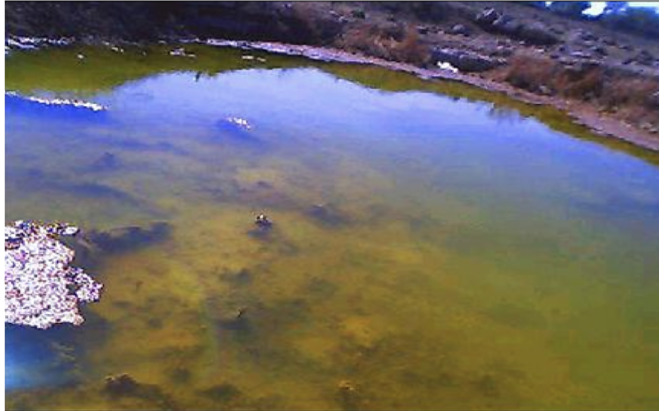
Chromium-contaminated pond with surface water contamination due to the discharge of large amounts of chromium-contaminated leachate

**Figure 7:**
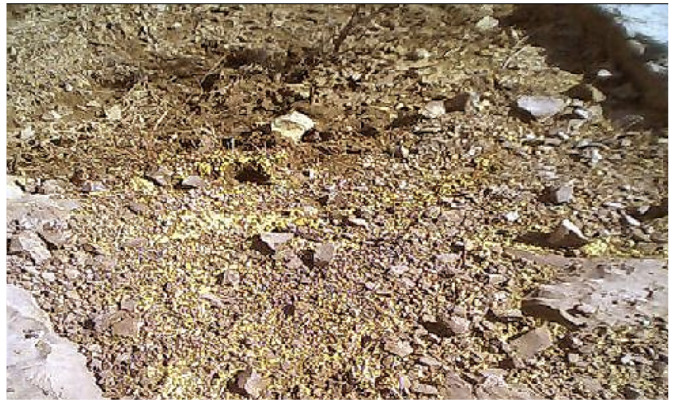
Chromium-contaminated pond with surface water contamination due to the discharge of large amounts of chromium-contaminated leachate

It is well known that the presence of Cr(VI) in concentrations above 0.5 mg/L impart a characteristic yellow color to the impacted water.^3^ At the study location, most of the seepage water coming out from the limestone fissures had a yellow color and was found to contain Cr(VI).

### Seasonal variation in chromium contamination in the impacted media

To identify the effect of seasonal changes in temperature and precipitation, sample collection and analysis were conducted in two different seasons, i.e., pre-monsoon (February–May) and post-monsoon (October–January). In these analyses, chromium levels showed a clear trend of seasonal variation *(Table 3).* In the trend analysis, surface water showed maximum contamination of Cr(VI) during the post-monsoon period (highest value of 22 mg/L) and a maximum of 2.53 mg/L during the pre-monsoon season. Thus, it can be concluded that Cr(VI) is being actively mobilized by percolating rainwater. The mine dewatering operation in the nearby limestone mines is contaminating the soil, surface and groundwater sources around the active mines.

**Table 3:** Concentration Increase in Chemical Species in Surface Water in Two Different Seasons

**Contaminant**	**Pre-monsoon mean (mg/L)**	**Post-monsoon mean (mg/L)**	**Increase by times**
Cr(VI)	0.51	14	26.45
pH	7.16	7.49	0.05
SO_4_^2−^	19.2	136.09	6.09
Chloride ion (Cl^−^)	17.08	19.7	0.15
Total hardness	131	317.78	1.43
Calcium(II) ion (Ca(II))	107	225	1.10
Magnesium (II) ion (Mg(II))	24	92.78	2.87
Alkalinity	90.6	123.89	0.37
Na(I)	81.8	1468	16.95

Two contaminants that showed significant differences in enrichment during the two seasons were Cr(VI) and sodium(I) ion (Na(I)). These two contaminants had a mean enrichment (taking all the samples together) of about 27 times for Cr(VI) and about 17 times in the case of Na(I) *(Table 3)* over pre-monsoon levels. The leachate showed a dominant presence of sulfate ions (SO_4_^2−^) along with the sodium and chromium ions. This observation was consistent with the fact that the originating industry of the dumped wastes produced sodium chromate and dichromate from chromite ore.

Comparison of the mean results of two seasons in the impacted surface water *(Table 3)* revealed that changes in the concentration, or enrichment in case of the pH, total hardness (TH), alkalinity, calcium, and magnesium, were small compared to the results for chromium and sodium. High concentrations of Cr and Na during the monsoon season indicate that the leached ions originate from the landfill.

### Statistical, enrichment and correlation analysis

The descriptive analysis of all samples collected during the post-monsoon season *(Table 4)* presents a relatively stable discharge condition prevailing at all the sampling locations. The low standard error, significant difference (SD) (except in case of Na(I)) and smaller values of kurtosis and skewness support this inference.

**Table 4:** Descriptive Summary of Surface Water in the Post-Monsoon Season

**Parameter**	**Cr(VI)**	**pH**	**SO_4_^2−^**	**Cl^−^**	**TH**	**Ca(II)**	**Mg(II)**	**Alkalinity**	**Na(I)**
Mean	14.00	7.49	136.09	19.70	317.78	225.00	92.78	123.89	1468.00
Standard error	3.51	0.12	32.48	1.70	33.49	16.03	19.79	4.70	376.48
SD	10.53	0.35	97.44	5.10	100.47	48.09	59.38	14.09	1129.43
Kurtosis	−1.71	1.37	−1.71	−0.22	−1.68	−1.10	−1.77	−0.73	−1.71
Skewness	−0.83	−1.31	−0.74	−0.94	0.75	−0.18	0.69	−0.23	−0.56
Minimum	0.01	6.75	4.50	10.60	215.00	155.00	35.00	100.00	12.00
Maximum	22.00	7.80	220.00	24.50	450.00	280.00	170.00	140.00	2560.00

Enrichment of the chemical species in the impacted water was compared to background values (uncontaminated control sample) prevailing in the surface water *(Table 5).* This comparison showed very high enrichment for Cr(VI), SO_4_^2−^, and Na(I) in the leachate impacted water. A significant increase in the TH, Ca(II), Mg(II) and a decrease in the alkalinity values was also noted, possibly due to dissolution of the bedrock limestone resulting in a mild increase in pH and a decrease in alkalinity (−38%). A very high enrichment in Cr(VI) (above 13999%), Na(I) (about 1531%) and SO_4_^2−^ (about 1411%) demonstrates the high degree of contamination in the Nadini-Khundini village in the Bhilai-Durg region.

**Table 5:** Enrichment of Chemical Species in Leachate of Impacted Water and Control Site

**Contaminants**	**Concentration in leachate water**	**Concentration in local surface water (control)**	**Percent enrichment in leachate**
Cr	14	<0.01	13999
pH	7.49	7.36	1.77
SO_4_^2−^	136	9	1411.11
Cl^−^	19	3	533.33
TH	317	169	87.58
Ca(II)	225	87	158.62
Mg(II)	93	12.2	662.29
Alkalinity	124	200	−38
Na(I)	1468	90	1531.11

As the waste dumped in the landfill is COPR, it is important to consider the impact of the pH of the percolating water. The solubility of Cr(VI) is controlled partially by Cr(VI)-hydrocalumite at pH >10.5 and by hydrotalcites at pH >8, in addition to adsorption of anionic chromate species onto inherently present metal oxides and hydroxides at pH <8. As pH decreases to <10, most of the Cr(VI)-bearing minerals will become unstable and their dissolution contributes to the increase in Cr(VI) concentration in a leachate solution.^27^

In the present study, the pH noted in rain and surface water ranged between 6.75–7.80 with a SD of 0.35, which indicates a neutral pH which is likely to make the Cr(VI)-bearing minerals in COPR very susceptible to aggravated leaching, leading to heavy loading of Cr(VI).

Even the groundwater used for drinking purposes, obtained from hand pumps in the affected village, showed the presence of Cr(VI) above the desired concentration of 0.05 mg/L according to the Indian standard for drinking water.^28^ Groundwater in this study refers to the hand pumps located in the village of Nadini-Khundini and a few locations in the nearby area. These hand operated borewells were shallow in depth (<10 m). Five borewells were studied in Nadini-Khundini. Four of the sampled borewells showed a low (0.04 mg/L Cr(VI)) to trace level of contamination. On the other hand, one particular hand pump, which lies topographically in the path of migrating mine water, showed a high level of contamination (0.26 mg/L Cr(VI)). This level is 5.2 times higher than the World Health Organization's permissible limit of 0.05 mg/L Cr(VI) in drinking water.^29^,^30^

The reported annual total rainfall in the studied area was 1288 mm.^31^ Over 80% of the total annual rainfall is received during the monsoon period between June to September. Hence, a considerable discharge of Cr(VI)-contaminated leachate takes place during the four months of the rainy season. A seasonal trend in the level of Cr(VI) is discernible in the results *(Table 6),* as chromium levels start to rise with the onset of monsoon and peak around the post-monsoon season. The ingress of rainwater in the landfill is the apparent reason for the generation of the contaminated migrating groundwater plume. The dilution effect appears to operate under the subsurface due to a mixing of the percolating water and existing groundwater channels that exist in the limestone bedrocks. Table 6 also shows that in contrast to the surface water, where the mobilization of Cr(VI) and other species are directly related to the rains during the monsoon season, the groundwater is high in contaminants during the post-monsoon season. Statistically, the results are characterized by lower SD, skewness and kurtosis values which signify a persistent contamination gradient across the contaminated region.

**Table 6:** Variation in Concentration in Groundwater Across Seasons

**Variable**	**Pre-monsoon**	**Post-monsoon**	**Change (%)**
Cr	0.26	0.04	−550.00
pH	7.25	7.03	−3.13
SO_4_^2−^	15.86	1.75	−806.29
Cl^−^	11.06	10.33	−7.07
TH	227	90	−152.22
Ca(II)	190	69.5	−173.38
Mg(II)	37	20.5	−80.49
Alkalinity	109.6	81.75	−34.07
Na(I)	16.6	3	−453.33

Results in mg/L, except for pH

Complete chemical analysis and statistical evaluation of the results were carried out. This analysis indicated that chromium was present in the form of chromium sulfate and sodium chromate.

The correlation analysis also showed extensive mobilization of chromium taking place during the monsoon season *(Table 7)* as evidenced by statistically insignificant (0.23) correlation of chromium with pH (suggesting a slightly acidic nature of the discharge) and a complete correlation (1 and 0.97) with sodium and sulphate, which underscores environmentally unaltered discharges. The correlation matrix in the post-monsoon season *(Table 8)* retains the high correlation of chromium with sodium and sulfate (0.99 each) and in addition correlates significantly with pH, TH, calcium, magnesium, and alkalinity. Based on this, a limited neutralization of the attendant acidity and almost no attenuation of the chromium or sulfate was discernible in the environment around the landfill.

**Table 7:** Correlation Matrix for Chemical Constituents of Contaminated Water in the Monsoon Season

	**Cr(VI)**	**PH**	**SO_4_^2−^**	**Cl**^−^	**TH**	**Ca(II)**	**Mg(II)**	**Alkalinity**	**Na(I)**
Cr	1.00								
pH	0.23	1.00							
SO_4_^2−^	**0.97**	0.24	1.00						
Cl^minus;^	0.45	0.04	**0.61**	1.00					
TH	0.28	**0.83**	0.31	0.32	1.00				
Ca(II)	0.42	**0.74**	0.45	0.39	**0.92**	1.00			
Mg(II)	−0.17	**0.55**	−0.14	0.01	**0.62**	0.27	1.00		
Alkalinity	**0.53**	0.22	**0.54**	0.19	0.25	0.17	0.28	1.00	
Na(I)	**1.00**	0.24	**0.97**	0.46	0.28	0.43	−0.17	**0.53**	1.00

**Table 8:** Correlation Matrix for Chemical Constituents of Contaminated Water in the Post-Monsoon Season

	**Cr(VI)**	**pH**	**SO_4_^2−^**	**Cl^−^**	**TH**	**Ca(II)**	**Mg(II)**	**Alkalinity**	**Na(I)**
Cr(VI)	1.00								
pH	**0.70**	1.00							
SO_4_^2−^	**0.99**	**0.73**	1.00						
Cl^−^	**0.73**	**0.67**	**0.73**	1.00					
TH	**0.65**	**0.70**	**0.64**	**0.74**	1.00				
Ca(II)	**0.51**	**0.78**	**0.53**	**0.68**	**0.86**	1.00			
Mg(II)	**0.58**	0.37	**0.54**	**0.55**	**0.81**	0.39	1.00		
Alkalinity	**0.62**	0.29	**0.68**	0.41	0.39	0.26	0.40	1.00	
Na(I)	**0.99**	**0.66**	**0.99**	**0.71**	**0.57**	**0.46**	**0.50**	**0.68**	1.00

### Impacts on watershed area

Groundwater near a landfill is mostly composed of percolated rainwater discharges from upgradient sources.^32^ Hence there is a mixing-dilution process that occurs between any leachate-polluted groundwater arising from the landfill and the groundwater in the vicinity of the landfill that ultimately will be transported off-site. This situation has been found to prevail at the study site as well.

Sampling area 3 was found to have a sizable number of inhabitants. It was also found that many people of the area work in the limestone quarry and are exposed to raw yellow colored leachates coming from the landfill area. The pond water, which was also contaminated with Cr(VI), was used for bathing and washing by the inhabitants. Questioning of local residents revealed that they were unaware of the possible ill-effects of the Cr(VI)-contaminated water, however, they reported adverse effects in exposed individuals and cattle.

**Figure 9:**
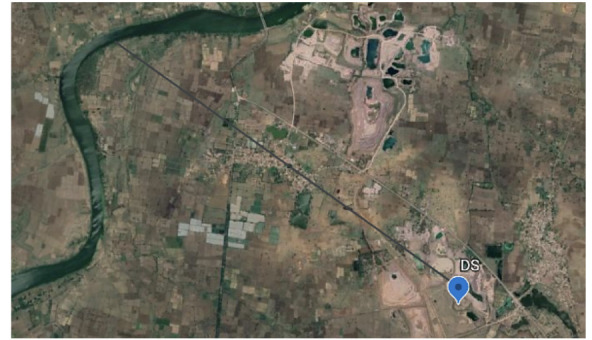
Drainage map of the impacted area. DS = Dumpsite, Aerial distance of the river 9.9 km.

The study region was found to be highly fractured and saturated with groundwater after a certain depth. In addition, the percolating rainwater migrates vertically downward from the landfill disposal area, which is located on the highest elevation point of the area. The maximum downward flows occur in the northern section of the landfill. The geographical flow of the water in the area is northward as evidenced by the regional topography, which culminates in the river Shivnath flowing in a north-easterly direction *(Figure 9).* Hence, it is evident that the surface water of the Shivnath River is at risk of contamination from the hazardous waste landfill at Nadini-Khundini village.

We noted the presence of stromatolites (a fossil relic from the marine environment) in the limestone deposits of the area. This feature lends credence to the layered depositional feature which is generally associated with a calm and shallow marine environment which might have prevailed in the region in the past geological history. This feature also indicates an absence of clay or eukaryotic organic depositions. This consideration is important for assessing the persistence of chromium in the contaminated groundwater plume.

## Discussion

In general, the chromite ore processing residue (COPR) waste are reported to be composed of calcium chromate (CaCrO_4_), calcium aluminochromate (3CaO•Al_2_O_3_•CrO_4_), tribasic calcium chromate (Ca_3_(CrO_4_)_2_) and ferric chromate (Fe(OH)CrO_4_). This waste is considered to be highly hazardous due to high residual contamination of hexavalent chromium. This process of sodium chromate manufacturing has been abolished in western countries, but is still practiced in countries like China, India, Russia and Pakistan,^33^–^35^ and therefore it is likely that they would experience related pollution.

An analysis of the eleven contaminated groundwater samples and the ratio of Cr(VI) to total chromium showed that almost all samples contained more than 99% of chromium in the form of Cr(VI) (*Table 9*). The results indicate favorable geochemical conditions for the mobilization and persistence of Cr(VI) and the absence of any inorganic or organic reductants in the landfill. The presence of Cr(VI) in the collected samples indicate that the study site an example of the hazardous waste mobilized contamination capable of causing potential adverse health effects noted at many other locations.^36^

**Table 9:** Ratio of Hexavalent Chromium Species to Total Chromium in Impacted Groundwater

**Sampled month**	**Total number of samples**	**Mean chromium level (mg/L)**	**Hexavalent chromium Cr(VI) (mg/L)**	**Trivalent Chromium Cr(III) (mean mg/L, by difference)**	**Ratio Cr(VI)/Total Cr**
July	11	0.51	0.50	0.01	0.99
February	11	1.51	1.49	0.02	0.99

### Soil analysis assessing natural attenuation potential

The USEPA has addressed chromium and its possible spread and impact on groundwater flow systems.^37^ It has further reported that natural attenuation of Cr(VI) will be subjected to a contaminated plume and local aquifer characteristics. This is due to the fact that the species of contaminants in the plume, and the presence of other ions, minerals and organic matter ultimately decide the fate of the contaminant in the environment. The soil matrix, abundance of probable reductants such as ferrous ions, organic matter, humic acid etc. can result in attenuation or reduction of highly oxidizing contaminants like Cr(VI).^38^

To assess the potential attenuation through neutralization or immobilization of the migrating plume of Cr(VI), a topsoil analysis was performed. Ten samples were collected from the topsoil around the landfill, and average results are presented in Table 10. These results demonstrate a limited availability of organic matter and acid neutralizing species in the soil around the landfill.

**Table 10:** Soil Quality of Study Area (Average of Ten Topsoil Samples)

**Serial No.**	**Variables**	**Nandini- Khundini Village**
1	Bulk Density (g/cm^3^)	1.23
2	Color	Brown
3	pH	7.21
4	Texture	Sandy loam
5	Bicarbonate (%)	0.05
6	Conductivity (μmhos/cm)	90
7	Chlorides (%)	0.01
8	Organic matter (%)	0.20
9	Available potassium (kg/ha)	171.50
10	Available phosphorus (kg/ha)	37.10
11	Available nitrogen (kg/ha)	110.60
12	Iron (%)	<1

As demonstrated in Table 10, the study location is significantly deficient in soil organic matter or iron compounds in soil. Hence, the surrounding soil cannot help with natural attenuation of Cr(VI) in the contaminated plume. This means that plume emissions generated in the study location are able to travel long distances without any appreciable attenuation of Cr(VI) species.

The question also arises whether the present location poses any other challenges compared to other limestone belts. Geologically, the limestone of the Durg district is pink limestone of the Raipur group, which is a part of the Chhattisgarh Supergroup formation. These rocks belong to the Proterozoic age.

Hydrogeologically, the aquifers of the area belong to a fissured media type, which is discontinuous and unconfined to semiconfined. They are generally restricted to weathered mantle and fractures and are in contact with the limestone. The aquifers extend as much as 150 m below ground level. The compounding issue here is that the area around the landfill in question is being actively mined by two adjoining and nearby stone quarries. The slope geometry in such mines is very steep (exceeding 45 degrees to almost vertical), and none have any provisions for berms or mine benches. This feature makes such quarries highly susceptible to instability, with tall face heights between 20 to 100 m. The percolating water seepage with high chromium content has caused problems for manual laborers working in the quarries, and to continue mining, chromium-laced seepage from all nearby quarries is pumped out by heavy duty pumps. This large discharge of water is a potential source of aquatic and environmental contamination.

### Advisability of siting of a hazardous waste landfill in an abandoned limestone quarry

An issue of concern is the lack of knowledge in the literature about dilution and dispersion in fractured rock and limestone systems.^37^ It is well known that reliable groundwater movement monitoring in fractured rock is not possible. The fundamental problem is that the flow occurs through fractures and cracks in the rock. Thus, monitoring wells spaced even a few feet apart may not be able to detect leachate transport through the bedrock unless they happen to intercept the fracture(s) that are principally responsible for leachate transport.^38^

The present study can help to address the advisability of siting a hazardous waste landfill in a stromatolitic-limestone terrain. The results show that the landfill in Nadini-Khundini is causing considerable discharge of Cr(VI) due to the improper construction of the landfill and facilitated by the apparent lack of appropriate standards during its construction.

The improperly designed hazardous waste landfill in Nadini-Khundini village in Bhilai and the siting of the landfill in limestone terrain and particularly in an abandoned limestone quarry is causing considerable discharge of Cr(VI) contaminated leachate. The landfill lacks any leachate collection or treatment mechanism. Furthermore, it has no surface water discharge mechanism after treatment and no monitoring of probable gaseous emissions. Other details of the waste type, volume, fill-area dimensions and related calculations are not available as the company is not willing to divulge this information. Entry to the landfill site is prohibited and secured by guards.

As seen in Figure 2, the constructed landfill has an estimated a perimeter of 400 m. Many nearby limestone quarries are >10 m deep. Thus, using an approximate depth of the landfill in the present study of 6–8 m, it is estimated that an approximate quantity of COPR waste dumped at the present site is 0.1–0.2 million tons. Chromite ore processing residue has been reported to have a residual chromium content of up to 50 000 mg/kg total chromium, half of which may occur as carcinogenic Cr(VI).^38^ Based on this, it can be estimated that the present site has approximately 5000–10 000 kg of total chromium out of which about 2500–5000 kg is hexavalent chromium. Such a huge quantity of hexavalent chromium in the COPR is likely to continue to contaminate the environment for an extremely long period of time.

The Siting Criteria in the Solid Waste Program (Ohio EPA) states that a sanitary landfill facility shall not be “located in a limestone quarry or sandstone quarry, unless deemed acceptable by the director”.^39^,^40^ A limestone quarry is defined as “an excavation resulting from a mining operation where limestone is the principal material excavated for commercial sale or use in another location. This term does not include excavations of limestone resulting from the construction of the sanitary landfill facility”. A sandstone quarry is defined as “an excavation resulting from a mining operation where sandstone is the principal material excavated for commercial sale or use in another location. This term does not include excavations of sandstone resulting from the construction of a sanitary landfill facility”.^41^ Sandstone and limestone quarries are generally thought to be semi-circular excavations whose walls are wholly composed of sandstone or limestone. The limestone or sandstone formations are generally fractured and saturated with water. Due to this, excavations may have to be pumped to prevent them from filling with water.

The Canadian Council of Ministers of the Environment states that an engineered hazardous waste landfill facility needs to be in a location with appropriate environmental conditions.^41^ Before deciding the location for a landfill, the geology, hydrology and hydrogeology of the area must be first considered and followed by meteorological, biological and other major environmental parameters.

The Indian hazardous waste rules do not specifically discuss siting criteria, or address the suitability of siting in abandoned limestone quarries.^42^,^43^ The Central Pollution Control Board guidelines propose the use of a liner system (single or double, depending on the rainfall, soil and water table).^44^ The present study suggests that this design would not likely succeed in stromatolitic-limestone terrains which receive high rainfall. The use of exhausted or abandoned quarries as hazardous waste landfills in India is likely to lead to ground and surface water pollution. Among the heavy metals, Cr(VI) is most mobile in an alkaline medium, thus the use of limestone quarries is expected to favor the mobilization of Cr(VI).^45^–^47^

The industry which generated the landfilled waste is situated in the industrial estate at Nandini Road, Bhilai, Durg district. As per the records of the Chhattisgarh Environment Conservation Board, the industry generates category A-5 (concentration limit: ≥ 50 mg Cr(VI)/kg) hazardous waste. The annual production of the COPR sludge from this industry is 4500 million tons per annum with a waste generation factor of 552.15 (quantity of hazardous wastes generated per unit (ton of product)).^48^ The present landfill site was approved by the state pollution control board known as the Chhattisgarh Environment Conservation Board. The present study estimates that the landfill contains around 0.1–0.2 million tons of COPR waste.

The present case study indicates that implementation of the design principles was not taken into consideration at the landfill. The chromite ore processing waste dumped at the studied site is classified as Schedule II hazardous waste as per the Hazardous and Other Wastes (Management and Transboundary Movement) Rules, 2016.^49^ The landfill contains thousands of tons of waste and the resulting leachate is causing long-term damage to residents living nearby and the aquatic environment. Direct damage to crops and aquatic life are apparent as the aquatic bodies impacted by the leachate are devoid of higher vertebrates. People exposed to these leachates have experienced sores and ulcers on their feet and hands. Potential impacts on the kidneys and digestive system have not been studied in detail. Residents in the study area also reported accidental poisoning of cows due to consumption of yellow colored discharge from the dumpsite. In most cases the consumption of undiluted discharge near the dumpsite was responsible for such deaths. The death of cows on accidental poisoning is similar to the cases reported by Eisler (1986).^50^

The present study adds to the literature regarding issues of hazardous waste disposal in limestone-rich regions. Based on the results of the present study, it can be concluded that the landfill in question is poorly designed and improperly sited in an abandoned limestone quarry. The large amount of rainfall during the rainy season and its entry in the landfill has resulted in the generation of huge quantities of leachates from this landfill.

Due to the surrounding alkaline medium, the condition at the site area favors the mobilization of Cr(VI). Hence the use of exhausted or abandoned quarries as hazardous waste landfill sites in India is likely to cause many further instances of ground and surface water pollution.

Persistence makes it possible for any contaminant to retain its chemical, physical or biological properties for a prolonged period. Persistence depends on the chemical structure, conditions in the aquifer conducive to degradation such as microbial population, nutrients available to support microbial growth, and the type and quantity of ions in the background groundwater. As described above, the stromatolitic-limestone geographical area is almost devoid of any organic deposition or clay in the area. Thus the Cr(VI) being released from the landfill is not likely to be adsorbed or transform to any other ionic state in such conditions. Hence the Cr(VI) generated from the site in Nadini-Khundini is likely to be transported in a highly toxic state for long distances to the boundary of the hydrological region, i.e., the Shivnath River.

The placement of a hazardous waste landfill in a limestone mine presents environmental and human health risks to the surrounding area. It is suggested that further delineation of the contaminated area and remediation and/or risk reduction actions to protect the public and natural resources from adverse health effects should be undertaken by the governmental authorities in this area. Remediation of the contaminates from this landfill is likely to be difficult and expensive. The appropriate authorities should consider the use of chemical reduction with sulfate-based materials as an immediate remediation step. This should be followed by bacterial bioremediation to ensure a safe and long-term solution.

## Conclusions

The present study aimed to identify and characterize the massive discharge of Cr(VI) from a hazardous waste landfill constructed in a former limestone quarry in the Nandini-Khundini area of Bhilai in central-east India. This landfill was officially permitted by the concerned authorities for disposal of wastes generated from a factory producing various chrome compounds. The raw material of the factory is chromite ore, and it is processed chemically to produce sodium chromate and dichromate. This process is known to produce considerable hazardous wastes containing a high amount of chromium and its intermediate compounds.

The maximum Cr(VI) concentration measured in the leachate was 1050 mg/L and 22 mg/L in the impacted surface water with a mean value of 14 mg/L in the post-monsoon season. Groundwater also showed the presence of Cr(VI) with the highest value of 0.26 mg/L in the pre-monsoon season. The average rainfall in the area was greater than 1288 mm, indicating significant percolation of rainwater in the landfill, and a high level of discharge of Cr(VI) from the landfill has been reported. Correlation studies in different seasons showed almost no attenuation of chromium or sulfate is taking place in the plume path around the landfill.

The present study also established that placing waste containing highly mobile compounds like Cr(VI) in a former limestone mine in a highly fractured stromatolitic-limestone environment is inappropriate. The geological environment in this area offers almost no resistance to the mobilization of Cr(VI). The absence of eukaryotic deposition has resulted in a minimal concentration of organic compounds in the soil or rocks of the study area. Therefore, highly toxic Cr(VI) is not being reduced to Cr(III) or other less harmful species. Furthermore, the drainage pattern in the study area indicates the possibility of translocation of contaminated discharge to the nearby river system on account of the favorable drainage pattern, non-reacting soil and prevailing lithology of the area. The lack of proper regulations, inadequate design, incomplete implementation of regulations, absence of monitoring and improper enforcement at the study site highlights the resulting adverse impacts on nearby residents and the environment.
